# Evolutionary and Functional Diversification of the Vitamin D Receptor-Lithocholic Acid Partnership

**DOI:** 10.1371/journal.pone.0168278

**Published:** 2016-12-12

**Authors:** Erin M. Kollitz, Guozhu Zhang, Mary Beth Hawkins, G. Kerr Whitfield, David M. Reif, Seth W. Kullman

**Affiliations:** 1 Toxicology Program, Department of Biological Sciences, North Carolina State University, Raleigh, North Carolina, United States of America; 2 Nicholas School of the Environment, Duke University, Durham, NC, United States of America; 3 Bioinformatics Research Center, North Carolina State University, Raleigh, North Carolina, United States of America; 4 Department of Biological Sciences, North Carolina State University, Raleigh, North Carolina, United States of America; 5 Department of Basic Medical Sciences, The University of Arizona College of Medicine, Phoenix, Arizona, United States of America; University of Texas Southwestern Medical Center, UNITED STATES

## Abstract

The evolution, molecular behavior, and physiological function of nuclear receptors are of particular interest given their diverse roles in regulating essential biological processes. The vitamin D receptor (VDR) is well known for its canonical roles in calcium homeostasis and skeletal maintenance. Additionally, VDR has received an increased amount of attention due to the discovery of numerous non-calcemic functions, including the detoxification of lithocholic acid. Lithocholic acid is a toxic metabolite of chenodeoxycholic acid, a primary bile acid. The partnership between the VDR and lithocholic acid has been hypothesized to be a recent adaptation that evolved to mediate the detoxification and elimination of lithocholic acid from the gut. This partnership is speculated to be limited to higher vertebrates (birds and mammals), as lower vertebrates do not synthesize the parent compound of lithocholic acid. However, the molecular functions associated with the observed insensitivity of basal VDRs to lithocholic acid have not been explored. Here we characterize canonical nuclear receptor functions of VDRs from select species representing key nodes in vertebrate evolution and span a range of bile salt phenotypes. Competitive ligand binding assays revealed that the receptor’s affinity for lithocholic acid is highly conserved across species, suggesting that lithocholic acid affinity is an ancient and non-adaptive trait. However, transient transactivation assays revealed that lithocholic acid-mediated VDR activation might have evolved more recently, as the non-mammalian receptors did not respond to lithocholic acid unless exogenous coactivator proteins were co-expressed. Subsequent functional assays indicated that differential lithocholic acid-mediated receptor activation is potentially driven by differential protein-protein interactions between VDR and nuclear receptor coregulator proteins. We hypothesize that the vitamin D receptor-lithocholic acid partnership evolved as a by-product of natural selection on the ligand-receptor partnership between the vitamin D receptor and the native VDR ligand: 1α,25-dihydroxyvitamin D_3_, the biologically active metabolite of vitamin D_3_.

## Introduction

Conjugated bile alcohols and bile acids (collectively referred to as bile salts) are multi-functional end products of cholesterol metabolism. They function as water-soluble amphipathic detergents that facilitate the solubilization and absorption of lipids, vitamins, and proteins in the small intestine [1]. All vertebrates synthesize bile salts, however studies have revealed substantial diversity in chemical structure across species. This diversity is hypothesized to be due to the evolution of an increasingly complex biosynthesis pathway that parallels vertebrate evolution [2,3].

**Fig 1:**
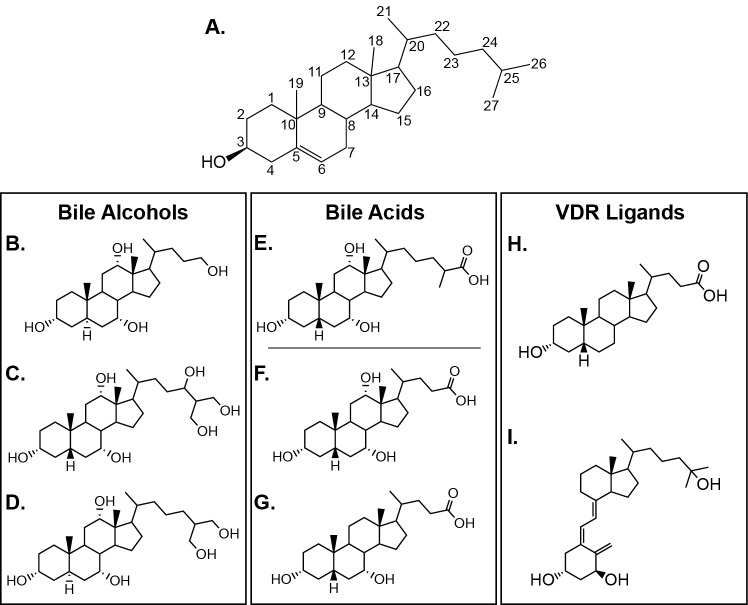
Chemical structures of vertebrate bile alcohols, bile acids, and vitamin D receptor ligands. (A) The structure of cholesterol, from which all bile acids and alcohols are derived. The left panel (B-D) depicts representative bile alcohols: (B) 5α-petromyzonol, a C_24_ bile alcohol that is a unique and minor component of the lamprey bile alcohol pool, (C) 5β-scymnol, the dominant bile alcohol of cartilaginous fish, and (D) 5α-cyprinol, the bile alcohol of zebrafish and other Cypriniformes. The middle panel (E-G) depicts representative bile acids: (E) the C_27_ trihydroxy bile acid that is the major bile acid of the Japanese medaka, and the two dominant C_24_ bile acids of vertebrates: (F) cholic acid (CA), and (G) chenodexocycholic acid (CDCA), the parent compound of LCA. The right panel depicts the two VDR ligands in this study: (H) lithocholic acid (LCA), the toxic metabolite of CDCA, and (I) 1α,25-dihydroxyvitamin D_3_ (1,25D_3_), the biologically active form of vitamin D_3_.

Bile alcohols are considered the most primitive class of bile salts due to the simplicity of the biosynthesis pathway and the fact that they are the dominant bile salt of basal vertebrates such as jawless, cartilaginous, and basal lobe-finned fish [2–4]. Bile alcohols retain all of the carbons from cholesterol (C_27_) and have a hydroxyl group on the terminal carbon of the cholesterol side chain (see Fig 1 for all chemical structures). C_27_ bile acids also retain all of the cholesterol carbons, however the terminal hydroxyl group has been oxidized to a carboxyl group. The C_24_ bile acids are considered to be the evolutionarily most recent class, and are utilized by more derived vertebrates such as birds and mammals. Unlike the C_27_ bile acids, the cholesterol side chain of C_24_ bile acids is truncated by three carbons. The most common C_24_ bile acids are cholic acid (CA) and chenodeoxycholic acid (CDCA).

Bile acids and alcohols are synthesized in the liver, conjugated, and released into the intestine following consumption of a meal. Over 95% of bile salts are actively reabsorbed at the end of the small intestine and returned to the liver for reuse [1,5]. The remaining bile salts enter the large intestine, where they undergo structural modifications mediated by anaerobic bacteria [6,7]. These bacterially synthesized bile acids are termed “secondary” bile acids in order to differentiate them from their primary counterparts that are synthesized in the liver. Of note, previous studies have demonstrated that bacteria do not modify bile alcohols, and thus they remain entirely primary [8,9].

While primary bile acids perform beneficial functions in vertebrate physiology, often their secondary products do not. Lithocholic acid (LCA, Fig 1H) is a toxic secondary bile acid produced by the bacterial 7-dehydroxylation of CDCA (Fig 1G) [6]. LCA is a known carcinogen that induces DNA damage through the production of reactive oxygen species and the formation of DNA adducts [10]. LCA acts as a tumor promoter by inhibiting DNA repair enzymes and promoting the proliferation of apoptosis-resistant cells [11]. Accordingly, an increased intestinal concentration of LCA as a result of a high fat diet is associated with an increased risk of colon cancer in mammals [12,13]. To counteract these detrimental effects, vertebrates have evolved detoxification mechanisms to protect against the toxicity of LCA and LCA metabolites. This process is mediated through the action of several nuclear receptors (NRs) that modulate transcription of metabolic phase I and phase II detoxification enzymes that drive the catabolism and elimination of LCA from the cell [6,14,15].

The farnesoid X receptor (FXR, NR1H4) [16], the pregnane X receptor (PXR, NR1I2) [14], and the vitamin D receptor (VDR, NR1I1) [15] are three NRs that mediate LCA detoxification. FXR and PXR are activated by a structurally diverse array of ligands, and display a high degree of cross-species variability in their ligand binding domains (LBDs) [4,17]. This variability is hypothesized to be a result of receptor-ligand co-evolution between the products of increasingly complex bile salt synthesis pathways and the LBD of both receptors [4,18,19].

However, no evidence of co-evolution has been identified between VDR and vertebrate bile salts [20,21]. In fact, studies have shown that VDR is subject to strong purifying selection as a well-conserved high fidelity receptor with narrow ligand specificity [18,22]. The primary endogenous ligand for VDR is the hormonally active metabolite of vitamin D_3_: 1α, 25-dihydroxyvitamin D_3_ (1,25D_3,_ Fig 1I). VDR is additionally activated by vitamin D_3_ metabolites and synthetic analogs, but is insensitive to other steroid hormones, vitamins, primary bile acids, and xenobiotics [15,22,23]. The canonical role of VDR is in regulating calcium homeostasis and skeletal maintenance, however additional non-calcemic functions have been identified, including roles in cell proliferation and differentiation, immune response, and neurodevelopment [24]. Like most NRs, VDR activation is initiated by ligand binding, which triggers a conformational change in the receptor and stimulates a strong association between VDR and the retinoid X receptor (RXR). The VDR-RXR heterodimer recognizes and binds to vitamin D response elements (VDREs) in promoter/enhancer regions of target genes and recruits members of the SRC/p160 family of nuclear receptor coactivators. Members of this family enhance transcription through chromatin remodeling and directing the progressive recruitment of additional co-integrator proteins to the transcription complex (see [25] for an in depth review of the VDR activation pathway). Gene transcription is initiated upon complex assembly.

Despite the narrow ligand specificity and high degree of sequence conservation exhibited by the receptor, LCA has been identified as a potent VDR ligand in mammals [15,26]. In fact, VDR is more sensitive to LCA than either PXR or FXR [15]. It has been proposed that LCA-mediated VDR activation evolved as an adaption by higher vertebrates to facilitate the detoxification of LCA [3,15,18,19]. Under this model, VDRs from basal vertebrates that utilize more primitive bile alcohols and acids are hypothesized to be insensitive to LCA. These species diverged from the vertebrate lineage prior to the evolution of the CDCA biosynthesis pathway, and thus do not synthesize the parent compound of LCA. In support of this theory, LCA has been demonstrated to activate mammalian VDRs in transient transactivation assays, but was incapable of activating VDRs from non-mammalian species such as the sea lamprey and zebrafish [15,21,22,27]. However, little functional work has been done to identify the molecular functions associated with the observed insensitivity of basal VDRs to LCA.

Here we characterize canonical NR functions of VDRs from species representing key nodes in vertebrate evolution and span a range of bile salt phenotypes. Species include the sea lamprey (*Petromyzon marinus*), a member of the most basal class of vertebrates (Agnatha, or jawless fish) [28] that utilizes mainly C_27_ bile alcohols, with a minor C_24_ alcohol component [2]. The little skate (*Leucoraja erinacea*) is a cartilaginous fish from the class Chondrichthyes (sharks, skates, and rays), which represents the earliest diverging lineage of jawed vertebrates [29]. Members of this class all utilize C_27_ bile alcohols [2]. The Senegal bichir (*Polypterus senegalus*) represents that most basal order of ray finned fish (Polypteriformes, class Actinopterygii), that diverged prior to the teleost lineage [29]. Bichirs synthesize a mixture of C_27_ bile alcohols and C_24_ bile acids (CA) [2]. The Japanese medaka (*Oryzias latipes*) is a teleost fish with a bile acid pool made up of roughly 90% C_27_ bile acids and 10% C_24_ bile acids (CA) [30]. A second teleost is the zebrafish (*Danio rerio*), a member of the order Cypriniformes. This order has lost the ability to synthesize bile acids, and instead synthesizes C_27_ bile alcohols [2]. To date, LCA has not been identified in any of these species [2,3,30]. Finally, we included human (*Homo sapiens*), a species that is known to synthesize LCA.

We hypothesized that LCA would not function as a VDR ligand in the majority of the species examined in this study. However, our results demonstrate that ligand affinity of VDR for LCA is highly conserved across extant species representing early origins of this receptor. These results suggest that VDR’s affinity for LCA was likely not driven through ligand-receptor coevolution as observed with PXR and FXR [4,18,19]. Furthermore, we found that NR activation steps subsequent to ligand binding varied between species, and the ability of the VDRs to respond to LCA may be driven through increasingly sensitive protein-protein interactions. We suggest that VDR-LCA partnership is likely an incidental by-product of natural selection on the ligand-receptor relationship between VDR and its native hormone (1,25D_3_). This relationship was likely later co-opted by higher vertebrates in order to mediate the detoxification of LCA.

## Materials and Methods

### DNA constructs

The pSG5, pVP16, and pET32a–VDR constructs were generated or obtained as previously described (see S1 Table for all GenBank accession numbers) [31–33]. All human RXRα (referred to in the manuscript as RXR), SRC-1, GRIP1, and ACTR constructs were a gift from Dr. Donald McDonnell (Duke University, Durham, NC). The luciferase reporters XREM-Luc, 5XGal4-TATA-Luc, and pRL-CMV luciferase control were obtained as described previously [31].

### Competitive Ligand Binding Assays

Radiolabeled 1α,25-dihydroxyvitamin D_3_ (1α,25-[26,27-^3^H]-dihydroxyvitamin D_3_, referred to as [^3^H]-1,25D_3_) was purchased from Perkin Elmer (Waltham, MA). The protease inhibitor cocktail was purchased from EMD Millipore (Billerica, MA). All cell culture media and other reagents were purchased from Corning Life Sciences (Corning, NY). Lithocholic acid (5β-cholanic acid-3α-ol, referred to as LCA) was purchased from Steraloids, Inc (Newport, RI).

Recombinant VDR and human RXR_WT_ lysates were prepared from transfected COS7 cells (American Type Culture Collection #CRL-1651) as described in detail previously [32,33]. Lysates were diluted 10-fold in ice-cold assay buffer (10 mM Tris HCl, 150 mM KCl, 1 mM EDTA, 0.3 mM ZnCl_2_, 5 mM DTT, and 1x protease inhibitor cocktail (100 μM AEBSF, 80 nM aprotinin, 5 μM bestatin, 1.5 μM E-64 (for cysteine proteases), 2 μM leupeptin, 1 μM pepstatin A), pH 7.5). 200 μL of lysate was transferred to each reaction tube that contained 4 nM [^3^H]-1,25D_3_ and 0–1 mM LCA. Reactions were shaken to mix and incubated for 18 hours at 4°C. Bound and free ligand were separated with the addition of 80 μL of ice-cold separation buffer (2.5% (w/v) activated charcoal, 0.5% (w/v) dextran, 100 mM Na_2_HPO_4_, 39 mM NaH_2_PO_4_, 150 mM NaCl, 15 mM NaN_3_, 10% gelatin, pH 7.0), and incubated at 4°C for 15 minutes with shaking every 5 minutes. Reactions were centrifuged at 5,000 x g for 5 minutes at 4°C, and 200 μL of the supernatant containing the VDR-bound ligands was removed for scintillation counting.

Total binding was determined using lysate from cells transfected with VDR, and nonspecific binding was determined using lysate from cells transfected with the empty pSG5 vector. Specific binding was determined by subtracting non-specific binding counts from the total binding counts. The concentration of LCA that inhibited 50% of [^3^H]-1,25D_3_ binding (IC_50_) was determined using nonlinear regression in Graphpad Prism 6. The receptor inhibition constant (K_i_), a measurement of VDR-LCA affinity, was obtained using the calculated IC_50_ and the previously calculated dissociation constant (K_d_) for each VDR and 1,25D_3_ [32,33] using the Cheng-Prusoff equation [34]. All assays were repeated three times with two technical replicates per LCA concentration.

### Transient Transactivation Assays

Lithocholic acid (LCA) was purchased from Steraloids as described above. 1α,25-dihydroxyvitamin D_3_ (1,25D_3_) was purchased from EMD Millipore (Billerica, MA). Cell culture media and other necessary reagents were obtained from Corning Life Sciences (Corning, NY).

HepG2 cells (American Type Culture Collection #HB-8065) were cultured in T75 flasks with vented caps (Corning Life Sciences, Corning, NY) using Minimum Essential Medium (MEM) with phenol red supplemented with 10% fetal bovine serum, 1 mM sodium pyruvate, and 1X MEM non-essential amino acids. Cells were maintained following standard protocols in a 5% CO_2_ incubator set at 37°C, and split when 80–90% confluent.

For the transient transactivation assays, HepG2 cells were seeded in 96-well tissue culture plates at a density of 2.5x10^4^ cells per well in complete phenol red-free MEM, and allowed to recover overnight. Transfections were performed the following day using Lipofectamine 2000 (Life Technologies, Grand Island, NY) as described previously [32,33]. For functional comparisons, 89.7 ng/well of full-length pSG5-VDR was co-transfected with 19.2 ng/well human XREM-Luc reporter and 4.5 ng/well of pRL-CMV as an internal luciferase control. Coregulator studies included the addition of 18.3 ng/well of human coregulators (pCDNA-RXR_WT_, pCDNA-RXR_AF2_, pSG5-SRC1, pSG5-GRIP1 or pSG5-ACTR) where indicated. Cells were dosed with 100 μM LCA in complete phenol-red free MEM 24 hours following transfection. Controls include 120 nM 1,25D_3_ as a positive control, and ethanol as a vehicle control. Twenty-four hours post-exposure cells were tested for luciferase activity using the Dual-Glo Luciferase Assay System (Promega Corporation, Madison, WI) according to the manufacturer’s protocols. Luciferase data were first normalized to the pRL-CMV internal luciferase control. LCA-mediated VDR transactivation (fold activation) was calculated as the ratio of the VDR luciferase response relative to the empty vector control. To calculate the impact of coregulators on VDR transactivation, VDR response in the presence of coregulators was normalized to VDR alone. All experiments were repeated 3 times and conducted as groups of 4 replicate wells. VDR response in the absence of coregulators was analyzed using a 1-way ANOVA followed by Dunnett’s multiple comparisons test. To examine the effects of different coregulators on VDR activation, data were analyzed using a 2-way ANOVA followed by Bonferroni’s multiple comparisons test in GraphPad Prism (GraphPad Inc, San Diego, CA).

### Mammalian 2-Hybrid Assays

LCA-mediated protein-protein interactions between the VDR orthologs/paralogs and essential nuclear receptor coregulators were analyzed using a mammalian 2-hybrid system (Clontech, Mountain View, CA). Prey constructs included full-length VDRs fused to the herpes simplex VP16 activation domain (pVP16-VDR). Bait constructs consisted of either full-length wild-type human RXR (pM-RXR_WT_), a truncated human RXR mutant (pM-RXR_AF2_), or the defined NR box of each member of the SRC/p160 family (pM-SRC1_241-386_, pM-GRIP1_479-767_, or pM-ACTR_392-1005_) fused to the yeast Gal4 DNA-binding domain. Reporters included the Gal4 luciferase reporter (5xGal4-TATA-Luc) and pRL-CMV as an internal luciferase control.

HepG2 cells were seeded into 96-well tissue culture plates as described above and transfected with Lipofectamine 2000 the following day. Each well received 33.6 ng pVP16-VDR, 33.6 ng pM-coregulator (where indicated), 126.6 ng 5XGal4-TATA-Luc, and 3 ng pRL-CMV. Cells were dosed with 100 μM LCA 24 hours post-transfection. Positive controls were treated with 1,25D_3_ and vehicle controls were treated with ethanol. Luciferase activity was measured 24 hours post-exposure using the Dual-Glo Luciferase Assay System described above. Raw luciferase data were normalized to the pRL-CMV luciferase control. VDR-coregulator response was normalized to the pVP16-VDR in the absence of bait constructs. Results were analyzed using a two-way ANOVA followed by Bonferroni’s multiple comparisons test in Prism (GraphPad Inc, San Diego, CA). All experiments were replicated 3 times and conducted as groups of 4 replicate wells.

### Bioinformatic Summary Analysis

In order to assess functional data in a global context, the mammalian 2-hybrid (M2H) and transient transactivation (TT) data for all VDRs tested were visualized as a heatmap using custom R code [35] as well as the R package Heatplus [36]. The Pickett Plot to the right of the heatmap indicates the presence/absence of co-regulators within each assay. The data were then normalized for each assay (row) across all eight VDRs to account for magnitudinal response differences. The resulting matrix was subjected to unsupervised, hierarchical clustering using Manhattan distance and complete linkage. Next, we performed bootstrap resampling over the assays according to presence/absence of each of the co-regulators to identify drivers of the overall cluster pattern as well as subclusters. For each of 10,000 bootstrap samples of assays, the accuracy was measured by counting the number of times the overall cluster pattern and species subclusters [lVDR, bVDR, sVDR, mVDRβ and zfVDRβ] and [mVDRα, zfVDRα, hVDR] were identical compared to the raw data. The lower the agreement with the original pattern, the higher the inferred importance of the assays to VDR functional similarity.

## Results

### VDR affinity for LCA is conserved across vertebrate evolution

**Fig 2:**
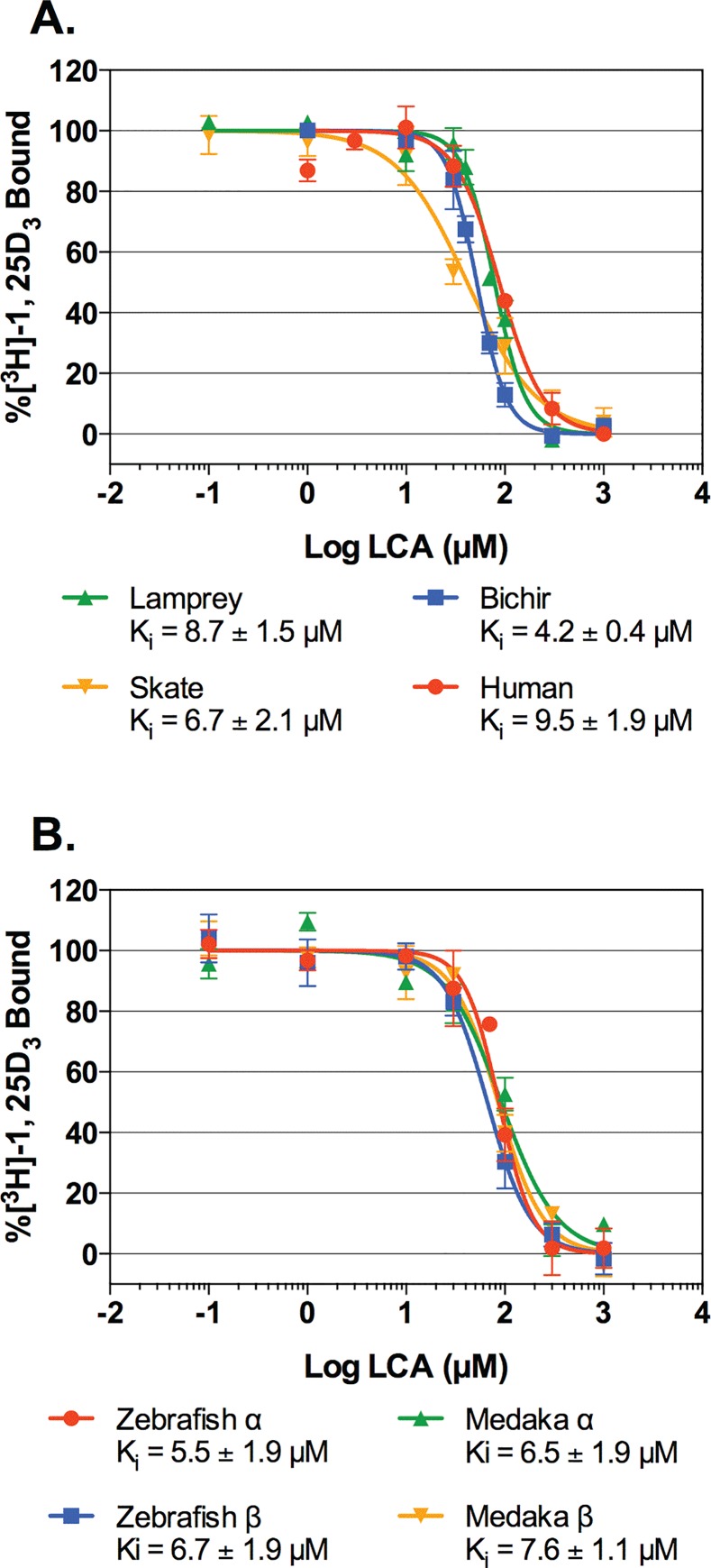
VDR competitive ligand binding assays for LCA in the presence of 1,25D_3_. Transfected cell lysates containing expressed VDR and human RXR_WT_ were incubated with a saturating concentration of 1,25D_3_ (4 nM) and a range of LCA concentration (0–1 mM). Reactions were incubated at 4°C for 18 hours. Bound and free ligands were separated as described in the Materials and Methods. The affinity of LCA for each VDR ortholog was determined by calculating the dissociation constant (K_i_) using the Cheng-Prusoff equation. The above graphs represent the combined specific binding data from three separate experiments (mean ± SEM).

Competitive ligand binding assays were utilized to analyze the ability of LCA to directly bind each VDR ortholog/paralog as a ligand. The results demonstrate that LCA functions as a specific and competitive ligand with the ability to displace 1,25D_3_ for VDR binding in all species tested (Fig 2A and 2B). Furthermore, all VDR orthologs/paralogs tested demonstrated similar binding affinities for LCA (K_i_ range = 4.2–9.5 μM), regardless of whether or not the parent bile acid of LCA (CDCA) was synthesized by that species.

### LCA does not activate non-mammalian VDRs in transient transactivation assays in the absence of exogenous coregulators

LCA was assessed for the ability to activate transcription of the VDR orthologs/paralogs using cell-based reporter assays in the absence of exogenous coregulators (Table 1). Similar to previous results from other studies [15,21,26], human VDR demonstrated significant transactivation in response to LCA (3.6-fold, p < 0.001). However, transactivation was not observed with the non-mammalian VDRs, with the exception of a small but statistically significant increase with zebrafish VDRα (1.8-fold, p < 0.05), a species that utilizes bile alcohols.

**Table 1:** VDR Transactivation mediated by 100 μM LCA

Species	Avg. Fold Activation ± SEM (n = 3)	*p*-value
Lamprey VDR	-	-
Skate VDR	-	-
Bichir VDR	-	-
Zebrafish VDRα	1.8 ± 0.3	< 0.05
Zebrafish VDRβ	-	-
Medaka VDRα	-	-
Medaka VDRβ	-	-
Human VDR	3.6 ± 0.4	< 0.001

### LCA induces VDR-RXR heterodimerization in select species

**Fig 3:**
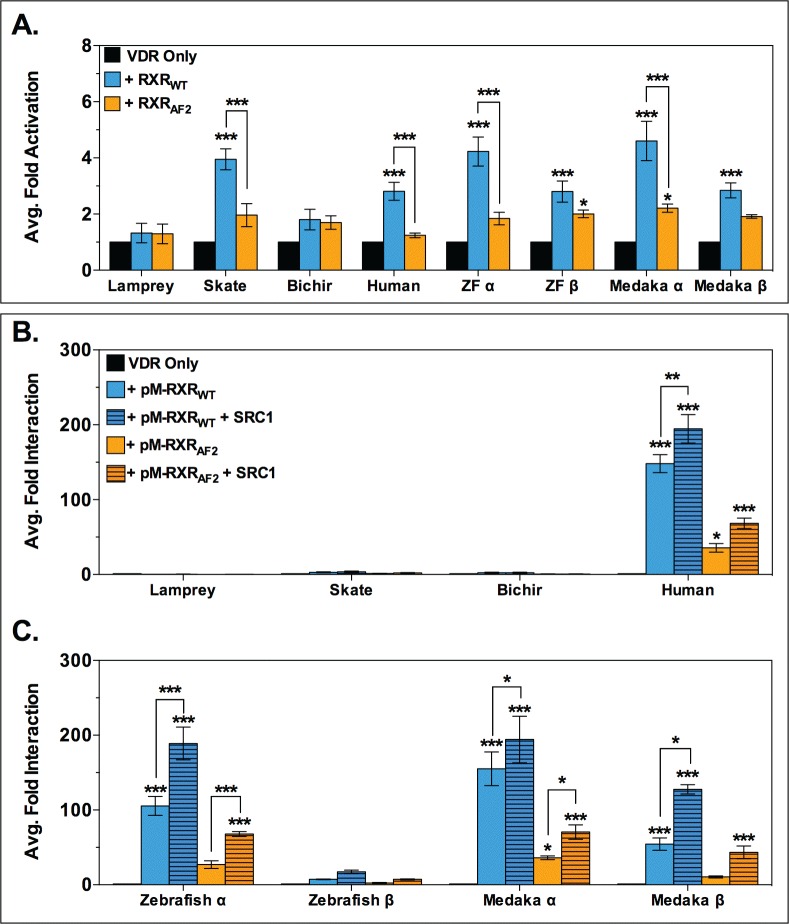
Analysis of VDR-RXR interactions in response to LCA. The top panel (A) illustrates the effect of exogenous human RXR on VDR transactivation in response to 100 μM LCA in transient transactivation assays. Human RXR constructs included wild-type RXR (RXR_WT_) and the RXR mutant lacking the c-terminal AF2 region (RXR_AF2_). VDR activation was measured via dual-luciferase assays and data were analyzed via 2-way ANOVA followed by Bonferroni’s multiple comparisons test. Asterisks over bars indicate a significant increase in VDR transactivation compared to the VDR only control (black bars), and asterisks over brackets indicate a significant difference in VDR activation in the presence of RXR_WT_ (blue bars) vs. RXR_AF2_ (orange bars): *** = p < 0.001, ** = p < 0.01, * = p < 0.05. Data are represented as the average fold activation normalized to VDR alone ± SEM (n = 3). The bottom panel (B and C) illustrates results from mammalian 2-hybrid assays that analyzed VDR-RXR heterodimerization in response to 100 μM LCA. VDR-RXR protein-protein interaction was measured via dual-luciferase assays, and analyzed via 2-way ANOVA followed by Bonferroni’s multiple comparisons test. Asterisks above bars represent a significant interaction compared to the VDR only control (black bars): *** = p < 0.001, ** = p < 0.01, * = p < 0.05. Asterisks over the brackets indicate that the addition of the SRC1 coactivator significantly increased VDR-RXR interaction (striped bars) compared to VDR-RXR interaction in the absence of SRC1 (solid bars). Data are represented as the average fold interaction ± SEM (n = 3).

To examine the effects of LCA on VDR-RXR interactions, transient transactivation studies were conducted supplementing assays with either wild-type human RXR (pCDNA-RXR_WT_) or a truncated human RXR mutant lacking the c-terminal AF2 region (pCDNA-RXR_AF2_). Data illustrated in Fig 3A demonstrate that assays supplemented with human RXR_WT_ facilitated and significantly enhanced transactivation of skate VDR, human VDR, and the VDRα and VDRβ paralogs from both zebrafish and medaka. Conversely, exogenous RXR_WT_ had no effect on either lamprey VDR or bichir VDR transactivation. Similar to previous studies with 1,25D_3_ [32,33], substituting RXR_WT_ with RXR_AF2_ attenuated VDR activation to near background levels.

In support of co-transfection studies, mammalian 2-hybrid (M2H) assays were conducted to determine if the increased VDR activation observed in the presence of exogenous human RXR_WT_ is due to LCA-mediated VDR-RXR protein-protein interactions (i.e. heterodimerization) (Fig 3B and 3C). Robust and significant VDR-RXR_WT_ interactions were observed for human VDR, zebrafish VDRα, medaka VDRα and VDRβ. These interactions were further increased with the addition of the nuclear receptor coactivator SRC1. By comparison, heterodimerization with RXR_WT_ was not observed with VDRs from the two basal vertebrates (lamprey VDR and skate VDR), the basal ray-finned fish (bichir VDR), and zebrafish VDRβ, both in the presence and absence of SRC1. Despite the absence of the AF2 region, a low level of VDR-RXR_AF2_ interaction was observed with human VDR and medaka VDRα, and addition of SRC1 to the assay enhanced VDR-RXR_AF2_ interactions with zebrafish VDRα, medaka VDRα and VDRβ, and human VDR.

### LCA-mediated VDR recruitment of the SRC/p160 family of nuclear receptor coactivators is limited to bony vertebrates

**Fig 4:**
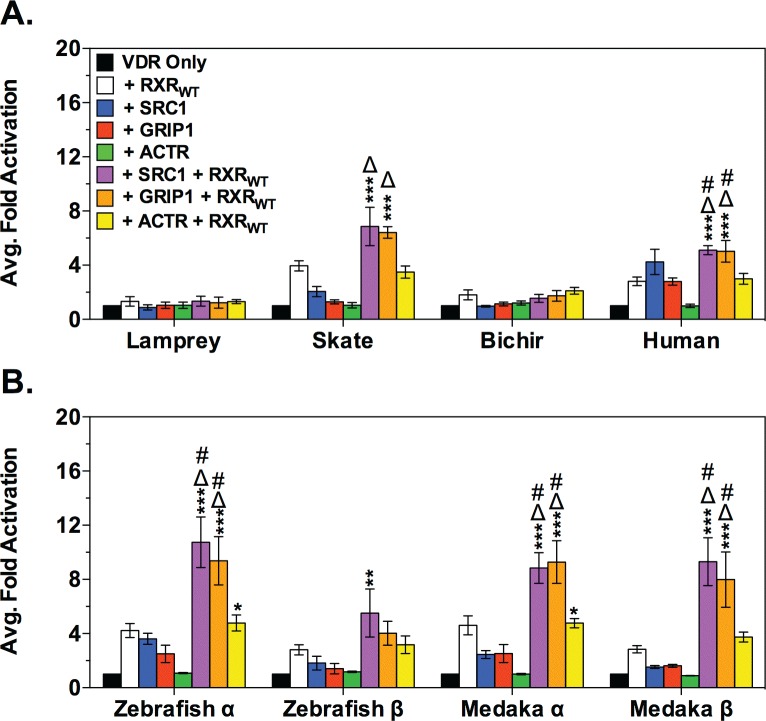
Analysis of LCA-mediated VDR transactivation with the SRC/p160 family of nuclear receptor coactivators. (A) and (B) illustrate the effects of exogenous human SRC/p160 nuclear receptor coactivators on VDR transactivation in response to 100 μM LCA in transient transactivation assays. The effect of the SRC/p160 coactivators on VDR transactivation was analyzed via 2-way ANOVA followed by Bonferroni’s multiple comparisons test. Asterisks represent a significant difference in transactivation compared to VDR in the absence of coactivators (black bars): *** = p < 0.001, ** = p < 0.01, * = p < 0.05. The Δ and # symbols indicate that the co-transfection of RXR_WT_ (#) or the indicated SRC/p160 coactivator (Δ) had a significantly greater effect on VDR transactivation than either the SRC/p160 coactivator or RXR_WT_ alone. Data are represented as the average fold activation normalized to VDR alone ± SEM (n = 3).

Transient transactivation assays were utilized to determine the potential of members of the SRC/p160 family of NR coactivators to enhance LCA-mediated VDR transactivation (Fig 4A and 4B). The members of the SRC/p160 family include the steroid receptor coactivator-1 (SRC1), the glucocorticoid receptor interacting protein-1 (GRIP1), and the activator of thyroid and retinoid receptor (ACTR) [37]. The addition of SRC/p160 co-activators did not significantly enhance VDR transactivation in any species tested in the absence of exogenous RXR_WT_. Co-transfection of RXR_WT_+SRC1 or RXR_WT_+GRIP1 significantly enhanced VDR transactivation zebrafish VDRα, medaka VDRα and VDRβ, and human VDR compared to the effects of either coregulator individually. Enhanced VDR transactivation was also observed with skate VDR and RXR_WT_+SRC1 or RXR_WT_+GRIP1, however the increase was not significantly different compared to the effect of RXR_WT_ individually. No change in transactivation was observed for either lamprey VDR or bichir VDR co-transfected with any of the SRC/p160 co-activators, either in the presence or absence of RXR_WT_.

**Fig 5:**
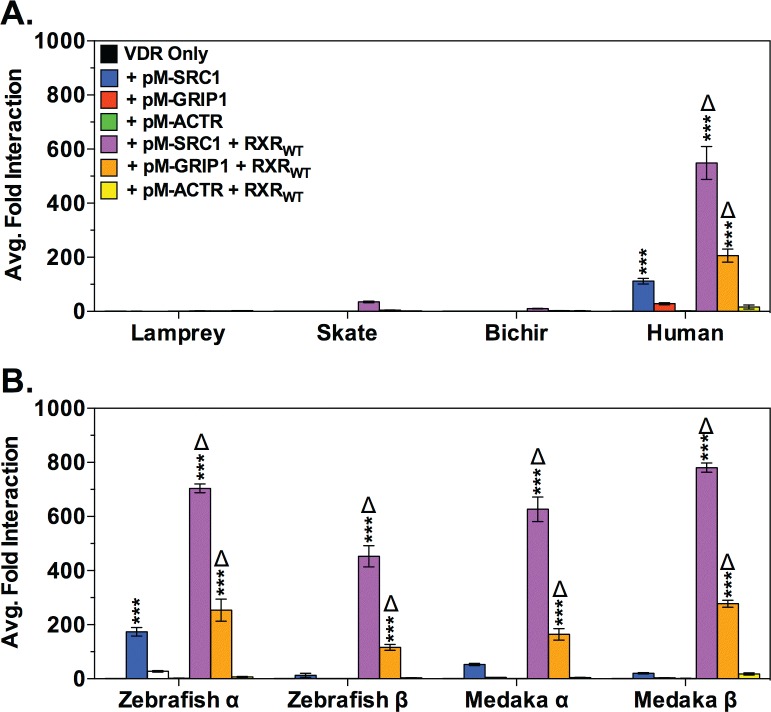
Analysis of protein-protein interactions between VDR and SRC/p160 nuclear receptor coactivators. (A) and (B) depict the results of mammalian 2-hybrid assays that assessed LCA-mediated protein-protein interactions between VDR and members of the SRC/p160 family of nuclear receptor coactivators. Significant VDR-SRC protein-protein interactions were analyzed via 2-way ANOVA followed by Bonferroni’s multiple comparisons test. Asterisks represent significant VDR-SRC interaction: *** = p < 0.001, ** = p < 0.01, * = p < 0.05. The Δ symbol indicates that the cotransfection of RXR_WT_ significantly increased VDR-SRC/p160 interaction compared to the absence of RXR_WT_. Data are represented as the average fold activation normalized to VDR alone ± SEM (n = 3).

Protein-protein interactions between VDR and the SRC/p160 coactivators were subsequently assessed using M2H assays in the presence and absence of exogenous RXR_WT_ (Fig 5A and 5B). In the absence of cotransfected RXR_WT_, only human VDR and zebrafish VDRα successfully recruited SRC1. Cotransfection of RXR_WT_ resulted in strong VDR-SRC1 interactions with human VDR, and the VDRα and VDRβ paralogs from medaka and zebrafish. Similarly, cotransfections with RXR_WT_ enhanced VDR-GRIP1 interactions with human VDR, and the medaka and zebrafish VDRα and VDRβ paralogs, however the interaction was attenuated compared to SRC1. No protein-protein interaction was observed with lamprey VDR, skate VDR, and bichir VDR with any of the SRC/p160 coactivators. No VDR-ACTR interactions were observed for any VDR tested.

### Evolving protein-protein interactions between VDR and nuclear receptor coregulators may be responsible for VDR gaining the ability to be transactivated by LCA

**Fig 6:**
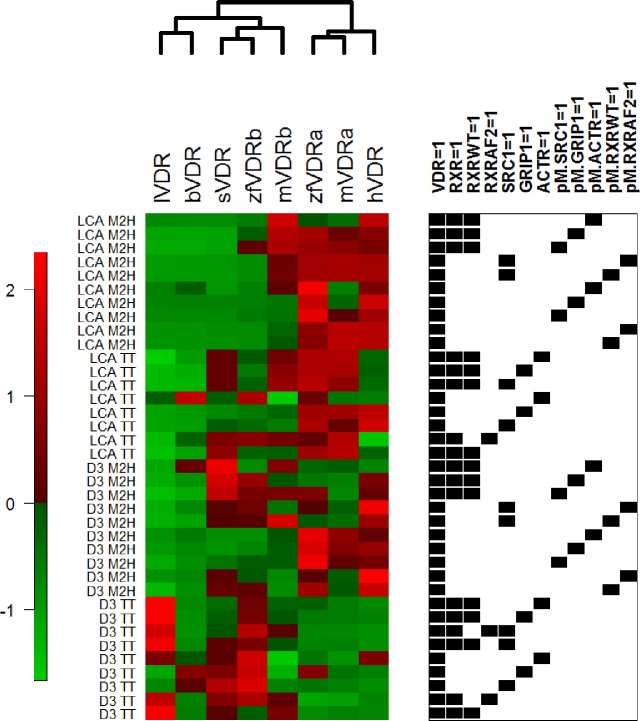
Heatmap depicting the results of the bioinformatic summary analysis. Analysis included mammalian 2-hybrid (M2H) and transient transactivation (TT) data for lamprey (lVDR), skate (sVDR), bichir (bVDR), human (hVDR), zebrafish (zfVDRa, zfVDRb) and medaka (mVDRa, mVDRb) in response to LCA and 1,25D_3_. The Picket Plot to the right of the heat map indicates the presence (black box) or absence (no box) of coregulators in each assay. Data were normalized for each assay (rows) across all species (columns) to account for magnitudinal response differences. The resulting matrix was subjected to unsupervised, hierarchical clustering using Manhattan distance and complete linkage. Bootstrap resampling was performed over the assays according to presence/absence of each of the coregulators to identify drivers of the overall cluster pattern as well as subclusters. For each of 10,000 bootstrap samples of assays, the accuracy was measured by counting the number of times the overall cluster pattern and species subclusters [lVDR, bVDR, sVDR, mVDRb and zfVDRb] and [mVDRa, zfVDRa, hVDR] were identical compared to the raw data.

Fig 6 provides a global, multispecies context for VDR functional assays using both LCA and previous data with 1,25D_3_ as ligands [32,33]. The data resulted in two empirical clusters of *C1* = [lVDR, bVDR, sVDR, and zfVDRβ, mVDRβ] and *C2* = [zfVDRα, mVDRα, hVDR]. These clusters were defined by responses across the entire assay set (rows), as annotated by the presence/absence of coregulators (indicated by presence or absence of black boxes in the Pickett Plot to the right of the heatmap in Fig 6). Cluster *C1* contains two separate subclusters. The first subcluster is composed of lamprey and bichir, which are defined by a low level of activity across the majority of assays with both ligands. The second *C1* subcluster contains skate VDR and the zebrafish and medaka VDRβ paralogs. Medaka VDRβ is on a separate branch from skate VDR and zebrafish VDRβ. This separation is most likely due to similarities in protein-protein interactions shared between skate VDR and zebrafish VDRβ, while medaka VDRβ demonstrates a higher level of activity with both ligands. The zebrafish VDRα and medaka VDRα paralogs formed a tight subcluster in *C2*, with a closely associated branch containing human VDR.

The bootstrap permutation results showed *C1* to be considerably more stable than *C2*, with greater than 99% recapitulation rate across all assay permutations. For *C2*, this analysis highlighted the importance of co-regulators in driving the observed cluster pattern, especially RXR and SRC1. Beyond specific cofactors, these results suggest that the functional-assay based clustering of the two alpha variants with human in *C2* depends on VDR responses across two-hybrid assays using LCA as the primary ligand.

## Discussion

The evolution, molecular behavior, and physiological function of nuclear receptors are of particular interest given their diverse roles in regulating many biological processes, including bile salt homeostasis. The vitamin D receptor is a nuclear receptor that is well known for its canonical roles in calcium homeostasis and skeletal maintenance. VDR has received an increased amount of attention recently due to the discovery of its numerous non-calcemic roles, including the detoxification of LCA. The ligand-receptor partnership between VDR and LCA has been hypothesized to be an adaptation limited to higher vertebrates such as birds and mammals, however the functional history of this partnership has not been explored. Here we expanded on previous studies examining the agonist activities of LCA on VDRs from non-mammalian vertebrates in comparison to 1,25D_3_.

We hypothesized that LCA would not function as a VDR ligand in the majority of the species examined in this study. Previous studies have demonstrated that LCA is potent mammalian VDR ligand in transient transactivation assays [15,27]. However, LCA was incapable of activating VDRs from non-mammalian species including lamprey and zebrafish [21,22,38]. Data from these studies have lead to the hypothesis that LCA-mediated VDR activation evolved later in vertebrate evolution, and evolved as an adaptation by higher vertebrates as a protective mechanism against LCA toxicity [4,19]. Accordingly, VDR orthologs from basal species that diverged from the vertebrate lineage prior to the evolution of the CDCA biosynthesis pathway do not synthesize the parent compound of LCA [2,3], and thus would be insensitive to the secondary bile acid.

Contrary to our hypothesis, we have demonstrated for the first time that LCA is able to effectively compete with the native VDR ligand (1,25D_3_) for binding to all VDRs tested in our competitive ligand binding assays. This includes species from lineages that diverged before the evolution of the CDCA biosynthesis pathway [2,3], and thus would never encounter the secondary bile acid. Furthermore, VDR’s affinity for LCA appears to be highly conserved across extant species that are separated by large evolutionary distances. For example, jawless fish diverged from the vertebrate lineage over 500 million years ago [39], yet VDR from the sea lamprey exhibited an affinity for LCA that is comparable to human VDR (8.7 μM vs. 9.5 μM, respectively). Furthermore, in the presence of exogenous coactivators, we observed LCA-mediated VDR transactivation and protein-protein interactions in select non-mammalian species that utilize bile alcohols or alternative bile acids. The fact that VDR’s affinity for LCA is highly conserved in species that either diverged from the vertebrate lineage prior to the evolution of LCA or utilize more ancestral bile salts supports the notion that VDR’s affinity for LCA may not be related to the evolution of the bile acid synthesis pathway or the primary bile salt utilized by each species [2,3].

We hypothesize that the VDR-LCA partnership is a by-product of natural selection on the ligand-receptor partnership between VDR and 1,25D_3_. Our hypothesis is supported by the fact that VDR exhibits a conserved binding affinity for both LCA and 1,25D_3_ across vertebrate evolution. This includes extant ancestral VDRs that evolved prior to the C_24_ bile acid biosynthesis pathway that is dominant in birds and mammals [2,3,6]. Structural studies using both teleost and mammalian VDRs have demonstrated that both LCA and 1,25D_3_ bind to the same canonical ligand binding pocket (LBP) within the VDR ligand binding domain, but in opposite orientations [40–42]. LCA maintains the same hydrogen bond anchors as 1,25D_3_, with the exception that some LCA-VDR hydrogen bonds are mediated by a water molecule, which forms a weaker bond and may contribute to the lower affinity of LCA compared to 1,25D_3_. The majority of the hydrophobic and hydrophilic interactions between LCA and the residues lining the LBP are identical to 1,25D_3_ [41,42]. The structural similarities between VDR bound to both ligands, even in zebrafish (which utilize bile alcohols), suggests that the affinity of LCA for VDR is likely related to the affinity between VDR and 1,25D_3_. Recently, zebrafish VDRα has been reported to have a second LBP outside of the canonical LBP, although its physiological function is unclear [42,43]. Our observation of LCA binding in zebrafish or other basal species may be associated with LCA interactions within this structure or both LBPs. While it is noted that both LBPs can promote receptor stabilization and active receptor conformation the molecular and/or physiological functions of ligand interactions with the non-canonical LBP remains unclear. What is yet to be determined however is if the second non-canonical LCA binding site is specific for VDR-SRC2 interactions as observed in the crystal structure.

Thus, while the ability of VDR to bind LCA appears to be an ancient trait, our results suggest that LCA-mediated VDR transactivation is not universal. We have previously shown that 1,25D_3_ activates all eight VDRs tested in transient transactivation assays and that VDR from all species are capable of protein interactions with RXR and SRC/p160 coregulators in response to this endogenous ligand [32,33]. However, in our current study we only observe VDR transactivation by LCA with human VDR as well as a small but significant increase in zebrafish VDRα (Table 1) [44]. The lack of transactivation in basal species may be related to putative differences in ligand-mediated *apo* to *holo* receptor transitions, ultimately impacting protein-protein interactions between the VDR orthologs/paralogs and essential coregulators. Supplementation of coregulators facilitated moderate increases in receptor transactivation, however mammalian 2-hybrid studies demonstrated direct protein-protein interaction between VDR and RXR only for human, zebrafish, and medaka, with a preference for zebrafish and medaka VDRα paralogs. Mammalian 2-hybrid between VDR and the SRC/p160 coactivators exhibited a similar pattern with only human, zebrafish and medaka exhibiting any protein-protein interactions which were dependent upon co-expression of RXR. These results suggest that basal species may lack the ability to form transcriptionally active conformations with LCA. This is likely due to an inability of LCA to re-localize helices H2, H3, H11 and H12 which induces a structural transition that triggers a mousetrap-like mechanism and stabilizes ligand binding and co-regulator recruitment [45]. Future studies to determine the crystal structures of basal VDRs or zebrafish VDRβ complexed with LCA may be very informative with regards to revealing species-specific differences in VDR conformations associated the observed functional differences. Currently only two VDR-LCA structures deposited in the RCSB Protein Data Bank [46] (URL: www.rcsb.org). One is for a mammalian VDR (*Rattus norvegicus*, PBD ID: 3W5P) [41] and the other is zebrafish VDRα (PBD ID: 4Q0A) [42], both of which are activated by LCA.

Hierarchical clustering of VDR functionality using both LCA and 1,25D_3_ as ligands further supports the role of RXR and the SRC/p160 family as important drivers of LCA-mediated VDR response. Lamprey, skate, bichir, and the two teleost VDRβ paralogs form a subcluster in C1. Lamprey and bichir form a separate subcluster within C1, most likely due to their inactivity in response to LCA and low response to 1,25D_3_. The VDRα paralogs group with human VDR in an independent cluster from the VDRβ paralogs and basal VDRs. This is likely due to the fact that the VDRα paralogs were able to heterodimerize with RXR_WT_ and recruit coregulators similar to human VDR. The bioinformatic analysis suggests that coactivator interaction may have been an important driver in the evolution of LCA as a functional VDR ligand. Although VDR has maintained an affinity for LCA, the ability of LCA to mediate successful protein-protein interactions between VDR and essential coregulators in order to mediate a functional transcriptional response may not have developed until much later in vertebrate evolution, likely within Osteichthyes (bony fish), before the split between Actinopterygii (ray-finned fish) and Sarcopterygii (lobed-finned fish and tetrapods). The idea that the LCA-VDR partnership is a byproduct of VDR’s relationship with the native ligand is further supported by our previous work with 1,25D_3_ in non-mammalian vertebrates. We have previously observed that 1,25D_3_ only behaves as a full agonist for VDRs from more recent lineages. This observation was consistent with the notion that increasing sensitivities to coregulator interactions were influential in the ability of VDR to evolve a full agonist response to 1,25D_3_ [32,33]. The more robust protein-protein interactions between VDR and coregulators in response to 1,25D_3_ may have evolved to facilitate the ability of LCA, a much weaker ligand, to functionally transactivate the receptor [27].

It is possible that the lack of observed VDR interactions with RXR and the SRC/p160 coactivators in basal species is due to the use of human coregulator proteins in these studies rather than coordinating species-specific VDRs and their inherent coregulators. However, we know that RXR and the SRC/p160 coactivators are found in most vertebrates and pre-vertebrate chordates including amphioxus (*Branchiostoma floridae*, subphylum Cephalochordata) [47] and tunicates (*Ciona intestinalis*, subphylum Urochordata) [48] which suggests that these proteins are ancient, predate vertebrate evolution, and are well conserved (see S2 Table, S1 Materials and Methods, and S1 Fig). We also know that the protein sequences of RXR (S2 Table), and the NR box of the SRC/p160 coactivators (which functions as the NR receptor interaction domain), are well conserved across vertebrate evolution (S1 Fig), suggesting that NR-RXR-SRC interactions may also be ancient. Additionally, we have previously observed that direct VDR-hRXRα and hSRC/p160 interactions [32,33] can occur across all species tested in this study using 1,25D_3_ as a ligand. These observations indicate that indeed protein-protein interactions are possible between species-specific VDRs and human coregulators. Thus, we have not found evidence that human coregulators are differential in their actions with non-mammalian nuclear receptors, possibly due to the highly conserved nature of these proteins [31–33,49]. Conversely, not all VDR ligands may function similarly and promote cross-species protein-protein interactions. Thus it will be interesting to discern if LCA as a VDR ligand can facilitate transactivation and protein-protein interactions between VDRs and their endogenous coregulators within species tested in this study.

The evolution of the partnership between VDR and LCA may be analogous to that observed for the ligand-receptor partnership of two corticosteroid receptors: the glucocorticoid receptor (GR, NR3C1) and the mineralocorticoid receptor (MR, NR3C2). While both receptors currently maintain distinct signaling functions, they evolved from an ancestral corticosteroid receptor that was duplicated roughly 450 million years ago at the base of the jawed vertebrate lineage [50]. GR is activated by cortisol, while MR is activated by aldosterone, a hormone that is specific to tetrapods. To date, aldosterone has not been identified in lower vertebrates such as jawless fish, sharks, and teleost [51]. However, functional assays using basal MRs and an ancestral MR/GR created through gene resurrection, found that these MRs were activated by aldosterone, despite the fact that the hormone evolved later in vertebrate evolution, and thus is only present in higher vertebrates [51]. The authors argue that the ability of basal receptors to bind aldosterone is an exaptation that was later co-opted by tetrapods once they evolved the ability to synthesize the hormone.

Overall, our results suggest that while the affinity for LCA appears to be an ancestral non-adaptive trait, the ability of VDR to be activated by LCA may have evolved more recently. This evolution likely entailed structural modifications to VDR that resulted in ability of LCA to effect a ligand induced physicochemical shift of H12 within the AF2 transactivation domain in order to facilitate selective recruitment of co-activators in later vertebrates. The partnership between LCA and VDR in basal vertebrates is likely to be a by-product of natural selection on the ligand-receptor partnership between VDR and 1,25D_3_. We hypothesize the ability of VDR to be activated by LCA may be a side effect of this evolutionary process that was later co-opted by higher vertebrates in order to detoxify and eliminate LCA. Conversely, given that a second non-canonical LBP has been observed in zebrafish, a role of alternative ligands and/or functions of VDR in basal species is still plausible.

## Supporting Information

S1 Table GenBank accession numbers. (PDF)

S2 Table Sequence homology of the retinoid X receptor (RXR). The numbers in each table represent the percent sequence identity conserved between the two species. The full-length receptor is depicted in (A). The ligand binding domain is depicted in (B) and the DNA binding domain is depicted in (C). Sequences were identified through BLAST analysis and aligned using CLUSTALW as described in S1 Materials and Methods. GenBank accession numbers can be found in table B in S1 Table.
(PDF)

S1 Fig Sequence alignment of NR boxes of SRC1 and GRIP1. (A) Depicts the conserved sequence motif of the SRC/p160 NR boxes: L represents leucine, and X can be any amino acid. (B) And (C) depict the sequence alignments of the three NR boxes for SRC1 (B) and GRIP1 (C). Sequence conservation is indicated by symbols at the bottom of the alignments as well as color-coding of the amino acids. The numbers flanking each NR Box correspond to the amino acid location in the full-length sequence. Sequences were identified through BLAST analysis and aligned using CLUSTALW as described in S1 Materials and Methods. GenBank accession numbers can be found in S1 Table.
(PDF)

S1 Materials and Methods Sequence Homology. (PDF)
