# Supplementary material for: Evolutionary and Functional Diversification of the Vitamin D Receptor-Lithocholic Acid Partnership
Source: PLoS One. 2016 Dec 12;11(12):e0168278. doi: 10.1371/journal.pone.0168278 (PMC5152921; doi:10.1371/journal.pone.0168278)
Supplement: S1 Table — (PDF) [file pone.0168278.s001.pdf]

S1 Table. GenBank Accession Numbers.

A. GenBank accession numbers for VDRs, RXR, and coactivators used in this study.

**Name**	**Species**	**Accession Number**
Lamprey VDR	*Petromyzon marinus*	AY249863
Skate VDR	*Leucoraja erinacea*	KJ925051
Bichir VDR	*Polypterus senegalus*	KJ925050
Zebrafish VDRα	*Danio rerio*	KJ925048
Zebrafish VDRβ		KJ925049
Medaka VDRα	*Oryzias latipes*	EU403115
Medaka VDRβ		EU403116
Human VDR	*Homo sapiens*	NM_000367.2
RXRα		AAH63827.1
SRC1		AAI11534.1
GRIP1		XP_016868450.1
ACTR		AAH92516.1

B. GenBank accession numbers for the RXR homology alignment.

**Name**	**Species**	**Accession Number**
Sea Lamprey	*Petromyzon marinus*	ABC49726.1
Elephant Shark	*Callorhinchus milii*	XP_007901074
Spotted Gar	*Lepisosteus oculatus*	XP_006640727.2
Zebrafish	*Danio rerio*	AAC59720.1
Japanese Medaka	*Oryzias latipes*	XP_011480292.1
Human	*Homo sapiens*	AAH63827.1

C. GenBank accession numbers for the SRC-1 NR box homology alignment.

**Name**	**Species**	**Accession Number**
Spotted Gar	*Lepisosteus oculatus*	XP_006625826.1
Arowana	*Scleropages formosus*	KPP69137.1
Zebrafish	*Danio rerio*	XP_691744.5
Sheepshead Minnow	*Cyprinodon variegatus*	XP_015228304.1
Nile Tilapia	*Oreochromis niloticus*	XP_013120497.1
Mouse	*Mus musculus*	AAH68177.1
Human	*Homo sapiens*	AAI11534.1

D. GenBank accession numbers for the GRIP1 NR box homology alignment.

**Name**	**Species**	**Accession Number**
Elephant Shark	*Callorhinchus milii*	XP_007885118.1
Spotted Gar	*Lepisosteus oculatus*	XP_015209106.1
Arowana	*Scleropages formosus*	XP_018609501
Zebrafish	*Danio rerio*	AAI63724.1
Japanese medaka	*Oryzias latipes*	XP_004081302.2
Nile Tilapia	*Oreochromis niloticus*	XP_007885118
Mouse	*Mus musculus*	AAB61575
Human	*Homo sapiens*	XP_016868450.1
