# Supplementary material for: Evolutionary and Functional Diversification of the Vitamin D Receptor-Lithocholic Acid Partnership
Source: PLoS One. 2016 Dec 12;11(12):e0168278. doi: 10.1371/journal.pone.0168278 (PMC5152921; doi:10.1371/journal.pone.0168278)
Supplement: S2 Table — The numbers in each table represent the percent sequence identity conserved between the two species. The full-length receptor is depicted in (A). The ligand binding domain is depicted in (B) and the DNA binding domain is depicted in (C). Sequences were identified through BLAST analysis and aligned using CLUSTALW as described in S1 Materials and Methods. GenBank accession numbers can be found in table B in S1 Table. (PDF) [file pone.0168278.s002.pdf]

S2 Table. Sequence homology of the retinoid X receptor (RXR)

A. Full-Length sequence

	HUMAN	MEDAKA	ZEBRAFISH	GAR	SHARK	LAMPREY
**LAMPREY**	69	68	72	70	69	100
**SHARK**	79	78	78	81	100	
**GAR**	88	89	79	100		
**ZEBRAFISH**	76	78	100			
**MEDAKA**	83	100				
**HUMAN**	100					

B. Ligand Binding Domains (LBDs)

	HUMAN	MEDAKA	ZEBRAFISH	GAR	SHARK	LAMPREY
**LAMPREY**	85	85	86	86	86	100
**SHARK**	93	93	91	93	100	
**GAR**	94	97	91	100		
**ZEBRAFISH**	90	90	100			
**MEDAKA**	94	100				
**HUMAN**	100					

C. DNA binding domains (DBDs)

	HUMAN	MEDAKA	ZEBRAFISH	GAR	SHARK	LAMPREY
**LAMPREY**	92	93	91	93	92	100
**SHARK**	98	98	97	98	100	
**GAR**	98	100	97	100		
**ZEBRAFISH**	97	97	100			
**MEDAKA**	98	100				
**HUMAN**	100					
