# Supplementary material for: Evolutionary and Functional Diversification of the Vitamin D Receptor-Lithocholic Acid Partnership
Source: PLoS One. 2016 Dec 12;11(12):e0168278. doi: 10.1371/journal.pone.0168278 (PMC5152921; doi:10.1371/journal.pone.0168278)
Supplement: S1 Fig — (A) Depicts the conserved sequence motif of the SRC/p160 NR boxes: L represents leucine, and X can be any amino acid. (B) And (C) depict the sequence alignments of the three NR boxes for SRC1 (B) and GRIP1 (C). Sequence conservation is indicated by symbols at the bottom of the alignments as well as color-coding of the amino acids. The numbers flanking each NR Box correspond to the amino acid location in the full-length sequence. Sequences were identified through BLAST analysis and aligned using CLUSTALW as described in S1 Materials and Methods. GenBank accession numbers can be found in S1 Table. (PDF) [file pone.0168278.s003.pdf]

S3 Fig. Sequence alignment of the NR boxes of SRC1 and GRIP1.

A. NR Box: LXXLL

B. SRC1 NR Box alignments

	NR Box-1	NR Box-2	NR Box-3
Gar[1]	576-AKLGQLLDG-584	614-KILHRLLQD-622	718-QLLRFLLDT-726
Arowana[2]	576-KLLNQLLDS-584	621-KILHSLLQD-629	699-QLLRFLLDT-677
Zebrafish	551-PKLSQLLDG-559	589-KILHRLLQD-597	634-QLLRFLLDT-642
Sheepshead Minnow[1]	571-SRLNQLLDS-579	615-KILHRLLQD-623	671-QLLRFLLDT-679
Tilapia[2]	581-PRLNQLLDS-589	631-KILHRLLQD-639	686-QLLRFLLGT-694
Mouse	635-HKLVQLLTT-643	692-KILHRLLQE-700	753-QLLRYLLDK-761
Human	631-HKLVQLLTT-693	688-KILHRLLQE-696	747-QLLRYLLDK-755
	* ***	**** ***:	****:**..

C. GRIP1 NR Box alignments

	NR Box-1	NR Box-2	NR Box-3
Shark[1]	640-TKLLQLLIA-648	701-KILHRLLQD-709	755-ALLRYLLDK-763
Gar[1]	646-TKLLQLLTT-654	700-KILHRLLQN-708	756-ALLRYLLDK-764
Arowana[2]	635-TKLLQLLTT-643	694-KILHRLLQN-702	747-ALLRYLLDK-755
Zebrafish	628-TKLLQLLTT-636	684-KILHRLLQN-692	740-ALLRYLLDK-748
Medaka	647-TKLLQLLTT-655	699-KILHQLLQN-707	778-ALLRYLLDK-786
Tilapia[2]	640-TKLLQLLTT-648	701-KILHQLLQN-709	755-ALLRYLLDK-763
Mouse	639-TKLLQLLTT-647	688-KILHRLLQD-696	743-ALLRYLLDK-751
Human	639-TKLLQLLTT-647	688-KILHRLLQD-696	743-ALLRYLLDK-751
	******* :	****:***:	*********

Consensus key
* single, fully conserved residue
: conservation of strong groups
. conservation of weak groups
no consensus

[1]SRC1 and GRIP1 sequences were not available for all species examined in this study. Species that are phylogenetically close to those in this study were used as substitutes for the alignments. Gar (*Lepisosteus oculatus*) was substituted for bichir (*Polypterus senegalus*). Sheepshead Minnow (*Cyprinodon variegatus*) was substituted for medaka (*Oryzias latipes*) for SRC1, and shark (*Callorhinchus milii*) was substituted for skate (*Leucoraja erinacea*) for GRIP1. Shark sequences were not available for SRC1. Lamprey (*Petromyzon marinus*) sequences were not available for either SRC1 or GRIP1.

[2]Arowana (*Scleropages formosus*) and Tilapia (*Oreochromis niloticus*) were included for comparison. Arowana represents a basal teleost from the order Osteoglossiformes. Tilapia represents an advanced teleost from the order Perciformes.
