# Supplementary material for: Evolutionary and Functional Diversification of the Vitamin D Receptor-Lithocholic Acid Partnership
Source: PLoS One. 2016 Dec 12;11(12):e0168278. doi: 10.1371/journal.pone.0168278 (PMC5152921; doi:10.1371/journal.pone.0168278)
Supplement: S1 Materials and Methods — (PDF) [file pone.0168278.s004.pdf]

SI Materials and Methods

Sequence Homology

Vertebrate RXR, SRC1, and GRIP1 sequences were obtained through BLAST analysis of the National Center for Biotechnology Information (NCBI; Bethesda, MD. URL: www.ncbi.nih.gov/). RXR DNA-binding domains (DBDs) and ligand-binding domains (LBDs) were identified from the annotated NCBI sequences. The sequences of the non-mammalian SRC1 and GRIP1 coactivators were compared to the human sequences in order to identify the NR boxes of each SRC/p160 coactivator. Predicted amino acid sequences of full-length RXR and the NR boxes of SRC1 and GRIP1 were aligned using CLUSTALW (1) via the SDSC Biology Workbench (2). GenBank accession numbers for all RXR, SRC1, and GRIP1 are listed in S1 Table. The RXR results are in Table S2, and the SRC1 and GRIP1 results are in S3 Fig.

References

1. Thompson JD, Higgins DG, Gibson TJ. CLUSTAL W: improving the sensitivity of progressive multiple sequence alignment through sequence weighting, position-specific gap penalties and weight matrix choice. Nucleic Acids Res. 1994;22:4673-80.
2. Subramaniam S. The Biology Workbench--a seamless database and analysis environment for the biologist. Proteins. 1998;32.
